# Biodegradation of Methyl Tertiary Butyl Ether (MTBE) by a Microbial Consortium in a Continuous Up-Flow Packed-Bed Biofilm Reactor: Kinetic Study, Metabolite Identification and Toxicity Bioassays

**DOI:** 10.1371/journal.pone.0167494

**Published:** 2016-12-01

**Authors:** Guadalupe Alfonso-Gordillo, César Mateo Flores-Ortiz, Liliana Morales-Barrera, Eliseo Cristiani-Urbina

**Affiliations:** 1 Departamento de Ingeniería Bioquímica, Escuela Nacional de Ciencias Biológicas, Instituto Politécnico Nacional, Ciudad de México, México; 2 Unidad de Biotecnología y Prototipos, Facultad de Estudios Superiores—Iztacala, Universidad Nacional Autónoma de México, Tlalnepantla, Estado de México, México; 3 Laboratorio Nacional en Salud, Facultad de Estudios Superiores—Iztacala, Universidad Nacional Autónoma de México, Tlalnepantla, Estado de México, México; Universite Paris-Sud, FRANCE

## Abstract

This study investigated the aerobic biodegradation of methyl tertiary-butyl ether (MTBE) by a microbial consortium in a continuous up-flow packed-bed biofilm reactor using *tezontle* stone particles as a supporting material for the biofilm. Although MTBE is toxic for microbial communities, the microbial consortium used here was able to resist MTBE loading rates up to 128.3 mg L^-1^ h^-1^, with removal efficiencies of MTBE and chemical oxygen demand (COD) higher than 90%. A linear relationship was observed between the MTBE loading rate and the MTBE removal rate, as well as between the COD loading rate and the COD removal rate, within the interval of MTBE loading rates from 11.98 to 183.71 mg L^-1^ h^-1^. The metabolic intermediate tertiary butyl alcohol (TBA) was not detected in the effluent during all reactor runs, and the intermediate 2-hydroxy butyric acid (2-HIBA) was only detected at MTBE loading rates higher than 128.3 mg L^-1^ h^-1^. The results of toxicity bioassays with organisms from two different trophic levels revealed that the toxicity of the influent was significantly reduced after treatment in the packed-bed reactor. The packed-bed reactor system used in this study was highly effective for the continuous biodegradation of MTBE and is therefore a promising alternative for detoxifying MTBE-laden wastewater and groundwater.

## Introduction

Methyl tertiary butyl ether (MTBE) is an oxygenated chemical that is widely used as a gasoline additive to improve the octane number and increase the combustion efficiency of gasoline by supplying extra oxygen during the combustion process, thereby reducing carbon monoxide and volatile organic carbon (VOC) emissions from internal combustion engines and air pollution [1,2]. However, extensive MTBE use has resulted in frequent soil, surface water and groundwater pollution, mainly due to accidental fuel leakage during storage and transportation [3,4], thus raising serious concerns about human and environmental health.

At present, Mexico produces and imports MTBE to meet its demand [5,6]. There have been some reports about the presence of MTBE in the environment in Mexico. MTBE concentrations of 11.5 mg m^-3^ [7] and 4.4 ppb [8] in air were recorded at a service station and emissions of on-road vehicles, respectively. Similarly, maximum MTBE concentrations of 0.88 and 0.3 mg kg^-1^ were found in soils at oil distribution and storage stations, respectively [9,10]. Likewise, MTBE concentrations ranging between 4 and 87 mg L^-1^ were found in groundwater near gas stations, but it was not detected in any of the 33 monitored drinking water wells nearby [11]. There is no legislation in Mexico that regulates gasoline releases or that defines MTBE limits in groundwater; however, all reported concentrations are higher than the Environmental Protection Agency advisory limit of 20 μg L^-1^ [11].

The odor and flavor thresholds of MTBE range between 2.5 and 190 μg L^-1^ and between 2.5 and 680 μg L^-1^, respectively [12]; so that very low MTBE concentrations make water undrinkable [13]. Additionally, human exposure to MTBE may cause coughing, headaches, muscular aches, fever, nausea, vomiting, dizziness, sleepiness and skin and eye irritation [1,14]. Moreover, MTBE is a known animal carcinogen and a potential human carcinogen and genotoxin [3,15]. Furthermore, MTBE may induce damage to deoxyribonucleic acid (DNA) in the form of single-strand breaks, double-strand breaks and oxidative base modification [3]. Microorganisms may also be more sensitive to MTBE as compared with larger organisms, including vertebrates and invertebrates, which may potentially affect bioremediation processes, nutrient cycling and organic matter degradation [4]. Therefore, the remediation of MTBE-contaminated water and soil is currently recognized as crucial to the protection of aquatic and terrestrial environments, as well as to public health.

A number of treatment technologies have been used to remove MTBE from aqueous solutions, including air stripping, adsorption, advanced oxidation processes and biodegradation processes [3]. However, MTBE remediation is challenging because of its high degree of water solubility (49 g L^-1^) and very low Henry’s law constant (5.87 x 10^−4^ atm m^3^ mol^-1^ at 25°C), which hinder its partition from the liquid phase to the vapor phase and promote low adsorption rates onto solids [1,14]. Biodegradation processes are recognized as cost-effective and environmentally friendly options and are therefore considered emerging technologies for the detoxification of MTBE-contaminated water [3,16].

Even though MTBE is recalcitrant to biodegradation due to its ether linkage and tertiary carbon, a number of microorganisms can either partially or completely degrade MTBE under aerobic or anaerobic conditions [17,18]. Only a few pure bacterial cultures can use MTBE as a growth substrate, while some others can degrade MTBE by co-metabolism, simultaneously consuming a different carbon source [19]. Furthermore, MTBE-degrading microbial consortia have attracted increased interest in recent years due to their species diversity. In a microbial consortium, one microbial population can breakdown a compound into a metabolite that can be further degraded by another microbial population, resulting in improved biodegradation efficiency and rates [17].

Existing biological methods for the biodegradation of MTBE involve the use of microorganisms in batch and continuous processes, using either suspended or immobilized microbial cultures. The main drawback associated with batch operation is that the initial MTBE concentration must be very low in order to prevent inhibition of microbial growth, which adversely affects MTBE removal rate [3,20]. It has also been suggested that the slow microbial growth rate and small amount of microbial biomass produced from MTBE utilization may cause low MTBE biodegradation rates [21]. In contrast, continuous culture systems for immobilized cells such as packed-bed biofilm reactors are intended to protect the microbial cells from stressful conditions and constitute an effective alternative to accelerating the rate of xenobiotic biodegradation [22]. Packed-bed biofilm reactors are also very useful in situations where the reactor capacity obtained by using freely suspended microorganisms is limited by biomass concentration and hydraulic retention time [23]. This is the case for slow-growing organisms such as the MTBE-degrading microorganisms, whose growth in suspension requires long residence times. Besides this, packed-bed biofilm reactors can be operated at high volumetric active-biomass concentrations that result in small reactor volumes, have an enhanced ability to perform well at low-influent substrate concentrations without the need to separate the biomass and the treated effluent, and provide improved productivity and stability [23–25]. Due to the above advantages, packed-bed biofilm reactors are extensively used in environmental biotechnology [23].

Here, we evaluated the capability of a microbial consortium isolated from gasoline-contaminated soil to biodegrade MTBE in a continuous up-flow packed-bed biofilm reactor. Additionally, we determined the metabolites produced during the MTBE degradation process using HPLC ESI-TOF-MS and measured the toxicity of the effluent from the bioreactor operated at different hydraulic retention times (HRT) using bioassays with organisms from two different trophic levels.

## Material and Methods

### Ethics statement

The studies conducted in this work did not involve endangered or protected species.

### Microorganisms

The natural microbial consortium used in this work was isolated previously from gasoline-contaminated soil collected in Mexico City, Mexico. This microbial consortium was obtained from the Culture Collection of the Microbiology Department of the National School of Biological Sciences, National Polytechnic Institute (IPN), Mexico City, Mexico, and designated as IPN-120526 [21]. The microbial consortium is mainly composed of five gram-negative bacterial species, which were molecularly identified by sequence analysis of the 16S rRNA genes and designated as *Pseudomonas delhiensis* IPN-TA, *Ochrobactrum* sp. IPN-TB, *Aminobacter aminovorans* IPN-TC, *Stenotrophomonas maltophilia* IPN-TD and *Sphingopyxis* sp. IPN-TE [21]. The IPN-120526 consortium and its bacterial constituents were cryopreserved with glycerol at -70°C.

### Culture medium

The liquid culture growth medium used throughout this work contained 1 g of KH_2_PO_4_·3H_2_O, 1 g of Na_2_HPO_4_, 0.1 g of MgSO_4_·7H_2_O, 1 g of NH_4_NO_3_, 1 mg of CaCl_2_·2H_2_O, 0.4 mg of FeSO_4_·7H_2_O, 0.1 mg of yeast extract, and 750 mg of MTBE (Sigma-Aldrich, St. Louis, MO, USA; 99.9% purity) per liter of distilled water [21]. All mineral chemicals and the yeast extract used for the preparation of the culture medium were of analytical grade and were supplied by J.T. Baker (Mexico) and BD Bioxon (Mexico), respectively. The liquid mineral medium was sterilized by autoclaving at 121°C for 20 min, whereas the MTBE was sterilized by microfiltration through 0.2 μm filters (Whatman). MTBE was added to liquid mineral medium following sterilization and cooling at room temperature.

### Bioreactor packing material

*Tezontle* was used throughout this work as a supporting material for the biofilm. *Tezontle* is an inert and highly porous, extrusive, volcanic, basaltic andesite scoria [26] with pH values near neutral, low cation-exchange capacity, good aeration and a moisture-holding capacity that is dependent upon particle diameter and provides a large contact surface [27,28]. Furthermore, *tezontle* does not contain toxic chemicals, is physically stable, can be sterilized and reused and is a low-cost material abundant in Mexico, and the presence of micropores in its structure allows for the establishment of microbial microcolonies [27–29]. Its usefulness as a packing material was demonstrated in different bioremediation processes [27,29–32].

In this study, *tezontle* stones were broken with a metal hammer to obtain fragments that were screened using sieves. The average equivalent diameter (*D*_*t*_) of *tezontle* stone particles used was 16.11 ± 2.35 mm.

### Packed-bed biofilm reactor

**Fig 1:**
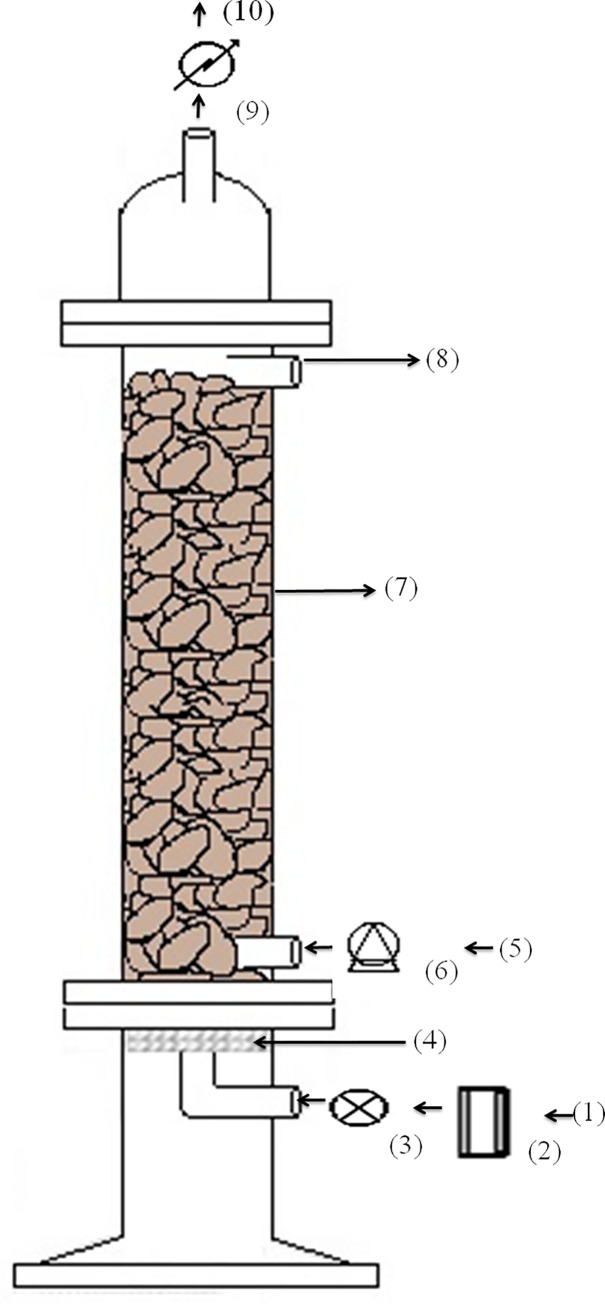
Schematic diagram of the packed-bed biofilm reactor [1) Air supply; 2) Air rotameter; 3) Air filter; 4) Porous glass diffuser; 5) Liquid input; 6) Peristaltic pump; 7) Packed-bed column; 8) Liquid output; 9) Condenser; 10) Exit gas].

All MTBE removal experiments were performed at room temperature (22 ± 2°C) in a lab-scale, packed-bed biofilm reactor system (Fig 1). The reactor system consisted of a feed tank, collecting tank, feed pump, gas rotameter, air filter, feed peristaltic pump, condenser and a reactor with inlet and outlet ports. The lid, base and column of the reactor were made of Pyrex glass. The cylindrical reactor column was packed with *tezontle* stone acting as the microbial biofilm support, and a mesh was located at the top of the packed-bed to prevent support movement. Discharge outlets for exhaust gas and liquid effluent were provided at the top of the lid and in the column of the reactor, respectively. The column was joined to the lid and base by metallic clamps and sealed with sterilizable silicon-rubber gaskets. At the center of the reactor base, there was a porous glass diffuser with a pore size of 30–40 μm. The packed-bed bioreactor was sterilized by autoclaving at 121°C for 20 min.

The air input was controlled through a pressure-regulating valve, and the airflow rate was measured with a Carboloy-float rotameter. A sterile airflow rate of 0.125 L min^-1^ was used. To reduce MTBE volatilization by air stripping and to encourage MTBE biodegradation, an ice water-cooled condenser was installed on the exit-gas line of the packed-bed reactor.

Once the reactor was assembled, the bulk density (*ρ*_*pb*_) and porosity (ε_*pb*_) of the packed bed were 0.75 g mL^-1^ and 62.6%, respectively, and the volumes of the total reactor (*V*_*T*_), packed bed (*V*_*pb*_) and the interstitial liquid (*V*_*L*_) were 250, 200 and 125.2 mL, respectively. The latter was used to calculate the HRT, as well as the volumetric loading rates (VLR), volumetric removal rates (VRR) and specific removal rates (SRR), of MTBE and chemical oxygen demand (COD).

### Kinetics of MTBE and COD removal

The packed-bed reactor was inoculated with 30 mL of a cell suspension of the microbial consortium containing 1.5 x 10^7^ CFU (colony-forming units) mL^-1^, which had been previously cultivated in culture medium. To ease microbial colonization of the porous *tezontle* stones, the reactor was operated in batch mode for 96 h under aerobic conditions and was subsequently drained and recharged with fresh culture medium, followed by initiation of a new batch culture. Repeated batch operation was continued until similar values of volumetric removal rates of MTBE and COD were obtained. Afterward, the packed-bed reactor was continuously fed with culture medium containing MTBE at a concentration (*C*_*oMTBE*_) of 750 mg L^-1^ and at a known feeding rate (F) until no significant change in MTBE and COD concentrations (± 5%) was detected in the liquid stream leaving the reactor (approximately at three HRTs: $HRT=\frac{V_{L}}{F}$), which indicated that a steady state had been reached. Several flow rates of culture medium ranging from 2 to 30.67 mL h^-1^, corresponding to HRTs from 62.6 to 4.1 h, volumetric loading rates of MTBE (*VLR*_*MTBE*_ = $\frac{FC_{oMTBE}}{V_{L}}$) from 11.98 to 183.71 mg L^-1^ h^-1^ and volumetric loading rates of COD (${VLR}_{COD}=\frac{FC_{oCOD}}{V_{L}}$) from 30.35 to 465.39 mg L^-1^ h^-1^ were assayed.

The MTBE-removal performance of the packed-bed biofilm bioreactor was evaluated using removal efficiency (*E*_*MTBE*_, %), volumetric removal rate (*VRR*_*MTBE*_, mg L^-1^ h^-1^) and specific removal rate (*SRR*_*MTBE*_, mg UFC^-1^ h^-1^), which in a continuous system operating at steady state can be calculated as follows:

$$E_{MTBE}=\frac{(C_{oMTBE}-C_{rMTBE})100}{C_{oMTBE}}=\frac{\left(VRR_{MTBE}\right)100}{VLR_{MTBE}}$$ 1)

$$VRR_{MTBE}=\frac{F\left(C_{oMTBE}-C_{rMTBE}\right)}{V_{L}}$$ 2)

$$SRR_{MTBE}=\frac{VRR_{MTBE}V_{L}}{X_{im}W}$$ 3)

where *C*_*oMTBE*_ and *C*_*rMTBE*_ are the MTBE concentrations (mg L^-1^) in the liquid entering and leaving the packed-bed biofilm reactor, respectively, *X*_*im*_ is the amount of biomass immobilized on the t*ezontle* stone particles (UFC g^-1^ dry *tezontle* stone) and *W* is the dry weight of *tezontle* stone particles (g).

The removal efficiency, volumetric removal rate and specific removal rate were also expressed in terms of the COD (*E*_*COD*_, *VRR*_*COD*,_ *SRR*_*COD*_). The removal rates and removal efficiencies are useful parameters to evaluate the biodegradation abilities of microorganisms [33].

To determine whether the observed MTBE removal was biological or abiotic, experiments without microbial biomass and with air supply to the reactor, as well as experiments without microbial biomass and without air supply, were conducted at the different feed flow rates tested. Biomass-free controls and without air supply were used to estimate abiotic MTBE removal by adsorption onto *tezontle* stones. In the biomass-free controls with air supply, any loss in MTBE content would be due to MTBE volatilization and/or adsorption onto *tezontle* stones.

The amount of MTBE adsorbed by the unit mass (dry weight) of *tezontle* stones, which represents the MTBE adsorption capacity (*q*, mg g^-1^), was calculated according to the following mass-balance relationship [34]:

$$q=\frac{\left(C_{oMTBE}-C_{rMTBE}\right)V_{L}}{W}$$ 4)

The MTBE removal experiments conducted in this work were reproducible within 5% error at most, and mean values from three replicates are reported herein. All data were statistically analyzed by one-way analysis of variance (ANOVA) with Bonferroni’s test (overall confidence level: 0.05) using GraphPad Prism software version 6.0c (GraphPad Software, Inc.).

### Toxicity bioassays

It is well known that the harmful effects of pollutants on environment and human health cannot be assessed by standard chemical analysis of environmental samples [35]. Contrastingly, toxicity bioassays play a relevant and crucial role in assessing the actual or potential impacts of chemical pollutants on the environment and human health [36]. Toxicity bioassays have the advantage of integrating the effects of all chemical compounds (including unknown substances, some of which may be breakdown products) contained in industrial effluents, treated effluents, environmental samples, etc., and take account of any additive and synergistic effects [37]. Furthermore, the use of bioassays involving organisms from different trophic levels is an efficient and essential tool for predicting environmental hazards to ecosystems [36,37].

In the present work, the toxic effects of the influent and effluents from the bioreactor were determined using bioassays with organisms from two different trophic levels. For these studies, influent and effluents samples were obtained from the MTBE-biodegradation experiments mentioned previously and used undiluted.

Microbioassays using bacterial species commonly found in soil and water were performed. The bacterial species used herein were as follows: *Escherichia coli* ATCC 25992, *Pseudomonas aeruginosa* ATCC 27853, *Enterococcus faecalis* ATCC 29212, and *Klebsiella pneumoniae* ATCC 700603, which were propagated according to the instructions provided by the American Type Culture Collection (ATCC). The bacterial toxicity tests were performed following the procedures outlined by Dutka and Kwan [38], with nutrient broth used as a control. The half maximal effective concentration (EC_50_) and the inhibition in the specific growth rate (μ) were used as criteria for evaluating toxic effects of influent and effluents from the packed-bed biofilm reactor on bacterial growth. Bacterial growth curves were determined by measuring the optical density (OD) at 590 nm using a spectrophotometer. The maximum biomass concentration reached by each bacterial culture was registered, and these data were used to estimate EC_50_ values. EC_50_ was defined as the MTBE concentration that caused a 50% reduction in the maximum biomass concentration between the control (bacterial species not exposed to MTBE and/or degradation metabolites during growth) and the studied samples. The specific growth rate (μ) of every bacterial culture was estimated as follows:

$$\mu =\frac{\ln X_{2}-\ln X_{1}}{t_{2}-t_{1}}$$ 5)

where *X*_*1*_ and *X*_*2*_ represent biomass concentrations at times 1 (*t*_*1*_) and 2 (*t*_*2*_) in the exponential growth phase, respectively.

The inhibition of specific growth rate was calculated according to the following equation:

$$\%\;inhibition=100-\left(\frac{\mu_{p}}{\mu_{c}}\right)\;100$$ 6)

where μ_p_ and μ_c_ represent the specific growth rates for the treated sample and for the control, respectively.

Phytotoxicity bioassays were also performed using seeds of *Lactuca sativa* as the biological test organism. These tests were conducted according to the procedures described by Young et al. [39], and distilled water was used as a control. The toxicity endpoints assessed were relative germination percentage (RG%), relative growth index (RGI) and germination index (GI), which were calculated as follows [39]:

$$RG\%=\left(\frac{Total\;number\;of\;germinated\;seeds\;\;in\;the\;sample}{Total\;number\;of\;germinated\;seeds\;in\;the\;control}\right)\;100$$ 7)

$$RGI=\left(\frac{Radicle\;length\;of\;the\;sample}{Radicle\;length\;of\;the\;control}\right)\;100$$ 8)

GI=(RG%)(RGI)100 9)

The RGI values were interpreted according to Young et al. [39] as follows: 1) Inhibition of root elongation: 0% < RGI < 80%; 2) no significant effects: 80% ≤ RGI ≤ 120%; and 3) stimulation of root elongation: RGI > 120%

In addition, the GI values were differentiated into three categories according to Zucconi et al. [40]: 1) GI values ≥ 80% indicated that there were no phytotoxic compounds in the sample or that they were present at very low concentrations; 2) values of 50% < GI < 80% indicated a moderate presence of phytotoxic substances in the sample, and 3) values of GI ≤ 50% indicated that there was a high presence of phytotoxic substances in the sample.

For each experimental condition in the toxicity bioassays, three replicates were prepared. Toxicity data were statistically analyzed by one-way analysis of variance (ANOVA) with Bonferroni’s test (overall confidence level = 0.05) using GraphPad Prism software version 6.0c (GraphPad Software, Inc.).

### Analytical techniques

#### Determination of attached viable biomass in the packed-bed bioreactor

The growth of the microbial consortium immobilized on porous *tezontle* stones was determined by viable cell counting (CFU g^-1^ porous dry *tezontle* stone). After reaching a steady-state condition at every hydraulic retention time assayed, we extracted the cells retained in weighed samples of porous *tezontle* stones. Each sample was washed and rinsed with a sterile solution of 0.85% NaCl in a vortex tube shaker until a clear extract was obtained. The extracted suspensions were collected and the total volume recorded. Serial dilutions of the resulting suspension were carried out, and 0.1 mL was inoculated in nutritive agar plates, followed by incubation at 37°C for 24 h. Finally, the number of colony-forming units (CFU) was recorded as CFU g^-1^ *tezontle* stone.

Having concluded the continuous culture runs, top, medium and bottom zones of the packed-bed biofilm reactor were sampled and bacterial cells were extracted and counted as described above. The bacterial strains were identified by their unique morphological characteristics.

#### MTBE and COD concentration determination

MTBE was extracted from influent and effluent samples according to an organic extraction method described by Karimi et al. [41] and quantified using a gas chromatograph (Agilent Technologies, model 6850) coupled to a mass spectrophotometer (Agilent Technologies, model 5975C). The gas chromatograph was fitted with a RTX-5MS diphenyl dimethyl polysiloxane (30 m long x 0.25 mm I.D. x 0.25 μm df) capillary column. Samples (1 μL) were injected into the column *via* a split injector with a split ratio of 100:1. The injector temperature was set at 250°C. The initial oven temperature was 40°C, subsequently increased to 50°C with a rate of 3°C min^-1^, and then moved to 58°C at a rate of 10°C min^-1^. The total run time was 4.13 min. Helium was used as the carrier gas at a flow rate of 0.7 mL min^-1^. Ionization source temperature, quadrupole filter temperature and electron ionization energy were set at 230°C, 150°C and 70 eV, respectively. Positive ions were analyzed in full scan mode from 35 to 400 m/z. A calibration curve was obtained using an MTBE standard (99.9% purity) from Sigma-Aldrich.

COD was quantified using the Hach method 8000 (range: 3–150 mg COD L^-1^) [42].

#### Analysis of MTBE biodegradation metabolites

The two most reported MTBE-biodegradation metabolites are tertiary butyl alcohol (TBA) and 2-hydroxy isobutyric acid (2-HIBA) [43–47], and their presence was evaluated in the effluent samples from the bioreactor following filtration through 0.7 μm filters (Whatman GF/F) and direct injection into the HPLC ESI-TOF-MS system. The gradient programs, as well as the ESI-TOF-MS and IT MS conditions used, were similar to those described by Rodríguez-Medina et al. [48].

HPLC analyses were performed using an Infinity UHPLC 1290 system (Agilent Technologies), equipped with a binary pump and a Kinetex® 2.6 μm C18, LC column 150 x 2.1 mm (Phenomenex). The conditions of the HPLC run were as follows: mobile phases: A: water/ACN 90:10 with 1% HCOOH, and B: ACN. The flow rate was set at 0.15 mL min^-1^. The gradient elution program was run as stated: 0 min, 5% B; 20 min 20% B; 25 min 40% B; 30 min 5% B; 35 min, isocratic of B 5% [48].

It was used an Agilent 6230 Accurate-Mass Time-of-Flight (TOF) LC/MS system equipped with an electrospray interface (ESI), model G1969-65338 from Agilent Technologies, with a mass range from 30 to 1000 m/z, operating in positive mode. The values of the ESI-MS parameters were as follows: gas heater temperature, 200°C; drying gas flow, 7 L min^-1^; nebulizing gas pressure, 22 psig; capillary voltage, 4500 V; fragmentor voltage, 200 V; and spectra rate, 1 Hz.

Data was processed using Agilent MassHunter Qualitative Analysis software (version B.06.00) with the “Generate Formulas” algorithm to determine the most likely formula from spectrum peaks. Additionally, identification of MTBE-biodegradation metabolites was performed by comparing their retention times and exact molecular weights with those of commercial standards of TBA (99.0% purity, Sigma-Aldrich) and 2-HIBA (99.0% purity, Sigma-Aldrich).

#### Observation of biofilm formation by scanning electron microscopy

For biofilm assessment in the packed-bed reactor, *tezontle* particles were collected, treated (biofilm was fixed with 2.5% glutaraldehyde, washed three times with phosphate buffer at pH 7, post-fixed with 1% osmium tetroxide, dehydrated with ethanol, dried, and covered with gold) and observed with a JEOL, JSM-5800LV scanning electron microscope at an accelerated voltage of 15 kV.

## Results and Discussion

### Consortium biofilm formation on *tezontle* stones

**Fig 2:**
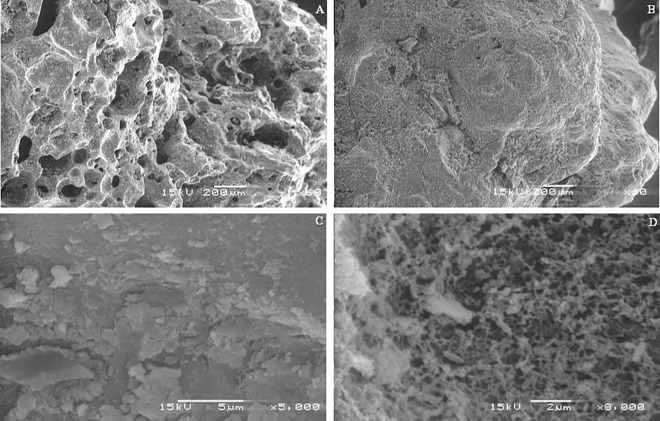
Scanning electron micrographs of *tezontle* stones (A) without biofilm (60×) and (B–D) with biofilm (60×, 5000×, and 8000×).

SEM analysis was performed in order to evaluate successful colonization of the microbial consortium on *tezontle* stones. Fig 2A shows a scanning electron micrograph of a *tezontle* control particle uncolonized by microbial consortium (control without biomass). The SEM micrograph revealed that *tezontle* particles had a rough and very porous surface texture, with irregular pores, enabling immobilization of microbial cells on different areas of the stone particles. Fig 2B–2D show the porous surface of a *tezontle* particle colonized with a biofilm exhibiting a complex architecture resembling a microbial mat. These observations indicated the presence of an abundant extracellular polymeric matrix that enclosed the cells in the biofilm. In this context, microbial biofilms contain extracellular polymers, such as polysaccharides, polyuronic acids, proteins, nucleic acids and lipids, that allow adhesion to solid surfaces, as well as combination and stratification with other microorganisms that do not have the ability to form biofilms [31,49,50].

For the biofilm on *tezontle* stones, microbial colonization and stratification might be organized according to the availability of oxygen and nutrients as previously suggested for other microbial biofilms [31,49]. We assumed that the microbial biofilm was organized based on of the ability and efficiency of the bacterial species to degrade MTBE and its metabolic intermediates. It is possible that bacterial species capable of performing the first round of MTBE-degradation reactions would be located in superficial areas of the biofilm, while bacterial species capable of utilizing the chemical compounds resulting from the initial MTBE degradation would be located in the lower strata [31].

### Biomass and MTBE biodegradation kinetics in the continuous up-flow packed-bed biofilm reactor operating in steady state

**Fig 3:**
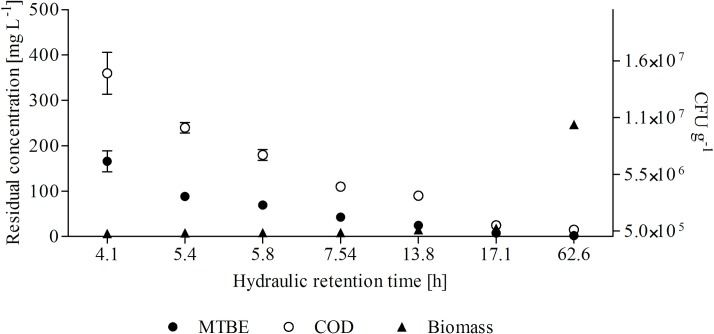
Biomass immobilized on *tezontle* stone particles and MTBE and COD concentrations of effluents at different hydraulic retention times.

Fig 3 displays the variations in the viable biomass attached to the *tezontle* stone particles, and the MTBE and COD concentrations in the liquid effluent with respect to HRT. Results showed a decrease in the bacterial population present in the microbial consortium, from about 10^7^ CFU g^-1^ to 10^5^ CFU g^-1^, as the HRT decreased from 62.6 to 4.08 h. This phenomenon may have been due to the shear stress caused by the fluid flow that tends to wash away the attached biofilm from the *tezontle* stones and increase as the fluid flow rate increases and the hydraulic retention time decreases. A similar trend was observed by Yañez-Ocampo et al. [32] in their studies on the removal of parathion and tetrachlorvinphos by a bacterial consortium immobilized on *tezontle* stones in a packed-bed reactor. To the best of our knowledge, no biomass results have been reported in the few studies available on MTBE biodegradation in packed-bed reactors operating in continuous mode at different hydraulic retention times.

Furthermore, results showed that upon installing a condenser, the experimental controls showed no significant losses of MTBE due to volatilization (≤ 2%). In addition, the adsorption capacity at equilibrium of MTBE onto *tezontle* stones was negligible (1.34 ± 0.46 mg MTBE g^-1^ *tezontle*), possibly due to its low log octane-water partition coefficient (log K_ow_ = 1.24) [51]. The low log K_ow_ of MTBE indicates that this xenobiotic is highly soluble in water and that its tendency to adsorb onto solids is low [14,52]. These observations suggested that neither MTBE volatilization nor adsorptive removal of MTBE by *tezontle* stones was significant in our experiments and that the major mechanism associated with MTBE removal observed in the MTBE-removal experiments with the microbial consortium was due to the biological activity of the microbial cells.

Furthermore, we observed that as HRT increased, the MTBE and COD concentrations progressively decreased until only low levels of MTBE and COD could be detected in the liquid effluent at HRTs greater than 5.8 h. At all HRTs assayed, the residual COD concentration was higher than the residual MTBE concentration. One explanation for this result may be that 1 g of MTBE is equivalent to approximately 2.7 g of COD [20,53].

**Fig 4:**
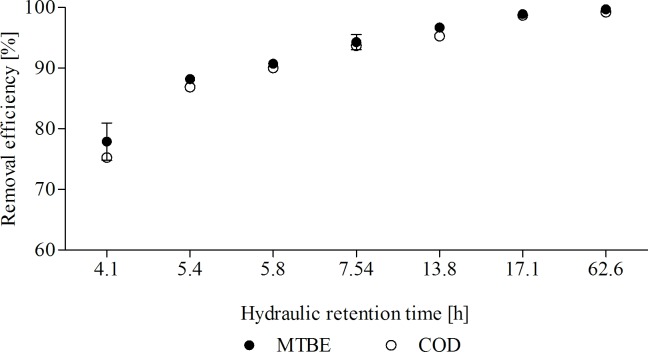
MTBE- and COD-removal efficiency at different hydraulic retention times.

The MTBE and COD removal efficiencies obtained at different HRTs are depicted in Fig 4. The MTBE removal efficiency increased from 77.87% to approximately 100% as the HRT increased from 4.1 to 62.6 h. Likewise, COD-removal efficiency increased with increasing HRT, reaching an efficiency of almost 100% at HRT values of 17.1 and 62.6 h. It should be noted from Fig 4 that MTBE and COD removal efficiencies greater than 90% were obtained at HRTs ranging from 5.8 to 62.6 h.

The increases in the MTBE and COD removal efficiencies along with the increases in HRT can be attributed to the increasing contact time between the microbial consortium and the culture medium, which favors the degradation of MTBE and other organic matter (e.g., MTBE-degradation intermediates).

In a study conducted in an up-flow, packed-bed biofilm reactor with a microbial consortium composed of three bacterial species, an unidentified gram-positive coccus, *Acinetobacter lwoffii* and *Bacillus* sp., Acuna-Askar et al. [22] reported that 53% and 70% of 150 mg MTBE L^-1^ was biodegraded at HRTs of 6 h and 12–24 h, respectively. Similarly, Bianchi et al. [54] reported 99.98% removal of 27.8 mg MTBE L^-1^ by a microbial consortium formed by *Alcaligenes* sp., *Pseudomonas* sp. and *Microbacterium* sp. in an up-flow, fixed-bed reactor operating at a HRT of 5 h. A microbial consortium was capable of removing 50% of approximately 160 mg MTBE L^-1^ in an up-flow, packed-bed reactor running at a HRT of 80 h [20]. Likewise, *Mycobacterium austroafricanum* IFP 2012 exhibited an MTBE removal efficiency higher than 99% in a fixed-bed reactor fed with a solution containing 15 mg MTBE L^-1^ and at an HRT of 120 h [55].

The MTBE-removal efficiency achieved in this study at HRTs ranging from 5.8 to 62.6 h was higher or similar to those reported in the aforementioned studies. However, the MTBE concentration used in our experiments (750 mg L^-1^) that was almost completely biodegraded at HRTs greater than 5.8 h was much higher than the MTBE concentrations used in the aforementioned studies and higher than the MTBE concentrations commonly degraded by pure [56–59] and mixed [60–62] bacterial cultures.

At all HRTs tested in the present study, the MTBE removal efficiency was slightly higher relative to the COD removal efficiency (< 3%). The small differences observed between the MTBE and COD removal efficiencies may be due to the formation of MTBE-biodegradation intermediates, the most common of which are TBA and 2-HIBA [43–47]. However, the similarly high MTBE and COD removal efficiencies obtained at HRTs greater that 5.8 h suggested than the microbial consortium was able to metabolize these metabolites.

Because pollutant removal rates could change according to culture conditions, the effect that volumetric MTBE and COD loading rates have on volumetric and specific MTBE and COD removal rates, respectively, was investigated.

**Fig 5:**
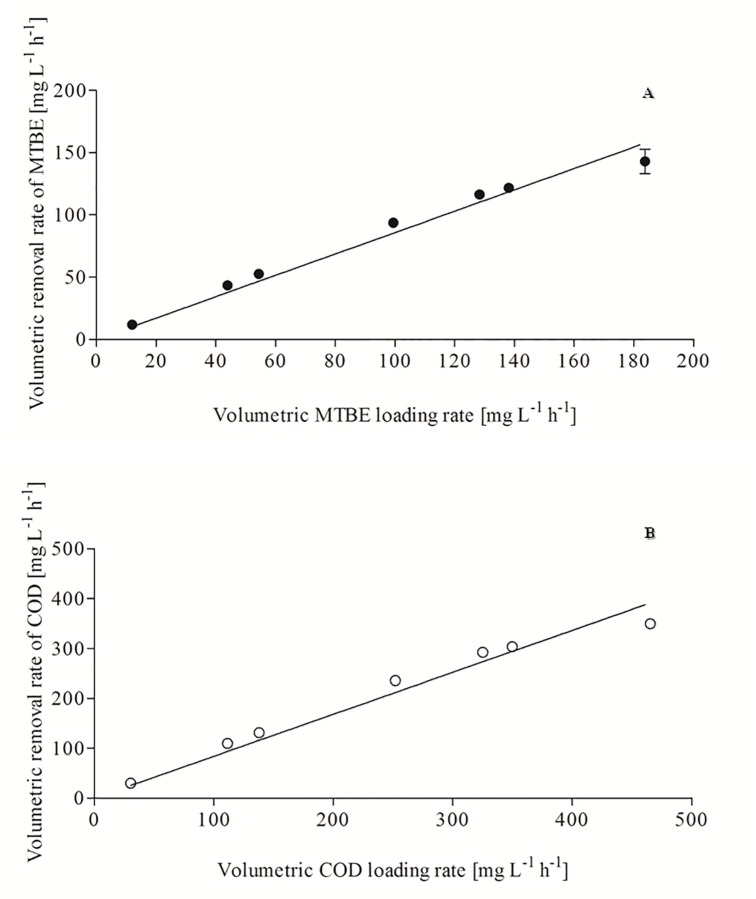
Dependence of volumetric removal rate on volumetric loading rate. (A) MTBE; (B) COD.

Fig 5 shows the volumetric MTBE and COD removal rates evaluated at seven hydraulic retention times, from 62.6 h to 4.1 h, corresponding to volumetric MTBE-loading rates from 11.98 to 183.71 mg L^-1^ h^-1^ and volumetric COD-loading rates from 30.35 to 465.39 mg L^-1^ h^-1^. We observed that volumetric MTBE and COD removal rates varied almost linearly (determination coefficients: R^2^ ≥ 0.975), with maximum values of volumetric MTBE and COD removal rates of 143 and 350.27 mg L^-1^ h^-1^, respectively.

Among the few known processes for MTBE biodegradation in up-flow, packed-bed reactors, Bianchi et al. [54] reported an estimated volumetric MTBE removal rate of 5.56 mg L^-1^ h^-1^ at a volumetric MTBE loading rate of 5.6 mg L^-1^ h^-1^. In addition, Acuna-Askar et al. [22] reported estimated volumetric MTBE removal rates of 5.35, 8.25 and 15.68 mg L^-1^ h^-1^ at volumetric MTBE loading rates of 6.09, 9.83 and 25 mg L^-1^ h^-1^, respectively. Similarly, estimated volumetric MTBE removal rates of 1 mg L^-1^ h^-1^ and 0.124 mg L^-1^ h^-1^ at volumetric loading rates of 2 mg L^-1^ h^-1^ and 0.125 mg L^-1^ h^-1^ were reported by Waul et al. [20] and Maciel et al. [55], respectively.

**Fig 6:**
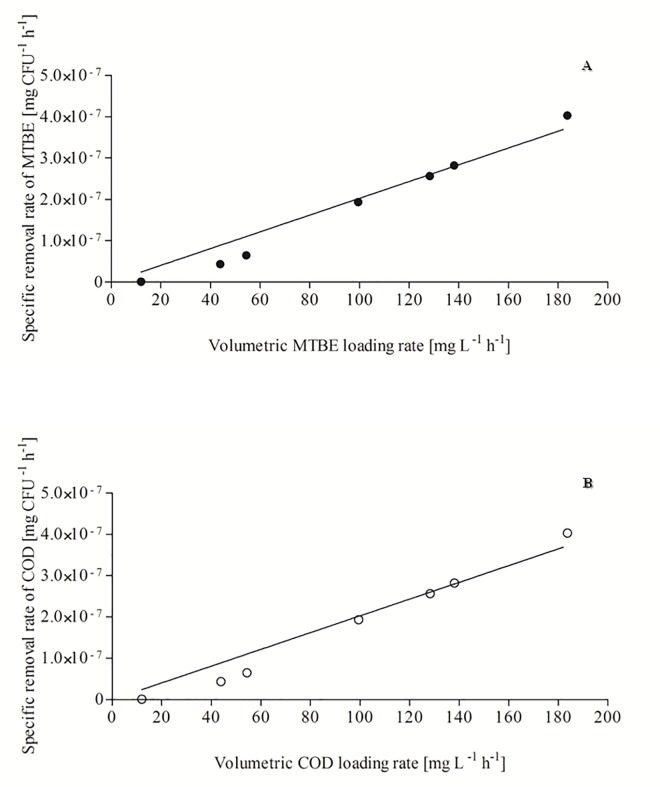
Dependence of specific removal rate on volumetric loading rate. (A) MTBE; (B) COD.

Fig 6 shows a linear dependence of the specific rate of MTBE removal on volumetric MTBE loading rate, as well as of the specific removal rate of COD on volumetric COD loading rate. The maximum values of specific removal rates of MTBE (4.03 x 10^−7^ mg MTBE CFU^-1^ h^-1^) and COD (9.34 x 10^−7^ mg COD CFU^-1^ h^-1^) were obtained at volumetric MTBE and COD loading rates of 183.71 and 465.39 mg L^-1^ h^-1^, respectively. It appears that no specific removal rates for MTBE biodegradation have been reported in the existing literature.

The fact that higher volumetric and specific MTBE removal rates and higher MTBE removal efficiencies were observed at higher volumetric MTBE loading rates in this study indicated that the microbial consortium has a remarkable ability to tolerate and degrade very high MTBE concentrations when cultivated in a packed-bed biofilm reactor and could therefore be useful for detoxification of MTBE-laden wastewater and groundwater.

It is worth mentioning that the packed-bed biofilm reactor operated for a period of more than 600 days and maintained high removal efficiencies and rates of MTBE and COD at HRT equal or higher than 5.8 h.

Table 1 shows the proportion of each bacterial strain present in the microbial consortium, both in the inoculum as well as in the top, middle and bottom zones of the packed-bed biofilm reactor. Results indicated that *Stenotrophomonas maltophilia* IPN-TD and *Sphingopyxis* sp. TE, which are the only two bacterial species present in the microbial consortium that are capable of degrading MTBE [21], as well as *Ochrobactrum* sp. IPN-TB, were the predominant bacterial species in the inoculum and in the three zones of the packed-bed reactor. Furthermore, *Pseudomonas delhiensis* IPN-TA was not detected in any of the three reactor zones, which indicates that this bacterial strain was washed away during the continuous culture runs.

**Table 1:** Relative abundance of bacterial strains that constitute IPN-120526 consortium in the inoculum and in the packed-bed bioreactor

Bacterial strain	Relative abundance (%)				
	Packed-bed reactor			Inoculum	
	Bottom	Middle	Top		
*P*. *delhiensis* IPN-TA	0.00 ± 00	0.00 ± 00	0.00 ± 00		7 ± 0.89
*Ochrobactrum* sp. IPN-TB	23.38 ± 5.19	22.91 ± 5.67	34.78 ± 17.6		29 ± 0.34
*A*. *aminovorans* IPN-TC	18.83 ± 9.74	12.32 ± 1.97	7.92 ± 6.36		14 ± 1.19
*S*. *maltophilia* IPN-TD	23.38 ± 5.19	38.18 ± 2.89	15.70 ± 6.18		36 ± 0.44
*Sphingopyxis* sp. IPN-TE	34.42 ± 20.1	26.60 ± 16.3	41.59 ± 17.8		14 ± 0.41

### Identification of MTBE degradation metabolites

Mass spectrometry analysis revealed that the exact molecular weight of the commercial standards of TBA and 2-HIBA was 75.0804 and 105.0436 g mol^-1^, respectively.

**Fig 7:**
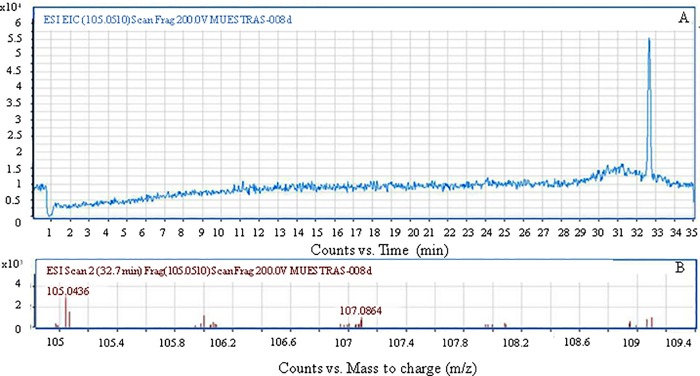
(A) Chromatogram and (B) ESI-TOF/MS spectrum of the effluent samples from the packed-bed reactor at HRT = 4.1 h.

In the present work, TBA was not detected by HPLC ESI-TOF-MS at any of the HRTs tested. Furthermore, 2-HIBA was also not detected at HRTs from 5.8 to 62.6 h; in contrast, at HRTs of 4.1 and 5.4 h, 2-HIBA was detected in the effluent from the packed-bed reactor, and its presence was confirmed with the corresponding standard in terms of retention time (32.735 min; Fig 7A), as well as with the exact molecular weight (105.0436 g mol^-1^; Fig 7B).

2-HIBA is the last MTBE-degradation intermediate containing the tertiary carbon atom, and according to proposed MTBE degradation pathways, the metabolic products generated from 2-HIBA can enter the central metabolism of MTBE-degrading bacteria [63]. Considering that no TBA or 2-HIBA was detected at HRTs from 5.8 to 62.6 h and that high MTBE and COD removal efficiencies were obtained in this HRT range, it is reasonable to assume that the microbial consortium used here is capable of assimilating the MTBE-degradation metabolic intermediates if enough contact time between the microbial cells and the circulating medium is allowed.

### Toxicity bioassays

We evaluated the effectiveness of the effluent-treatment system from the packed-bed biofilm reactor by assessing toxicity reduction with growth-inhibition tests using *Escherichia coli* ATCC 25992, *Pseudomonas aeruginosa* ATCC 27853, *Enterococcus faecalis* ATCC 29212 and *Klebsiella pneumoniae* ATCC 700603, as well as with the *Lactuca sativa* seed germination- and root elongation-inhibition tests.

**Fig 8:**
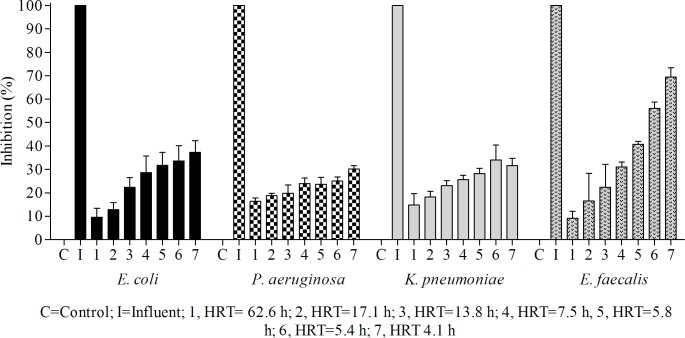
Toxicity tests on bacterial strains of influent and effluents at different hydraulic retention times.

Microbioassays using bacteria are increasingly applied to measure chemical toxicity in the environment. These bioassays are attractive because they are low cost, respond rapidly to toxicants, exhibit high sample throughput, require modest laboratory equipment and space, require low sample volumes, are portable and result in reproducible responses [64]. The results from the microbial growth-inhibition tests showed that the influent completely inhibited the growth rate of all bacterial species tested (100% inhibition in specific growth rate). In contrast, no inhibition was observed in the controls (Fig 8). The effluents inhibited the specific growth rate of all bacteria tested (Fig 8), and the percentage of inhibition increased as the MTBE concentration of the effluents increased, which occurred along with decreases in HRT (Fig 3). However, the influent caused a much stronger inhibitory effect on bacterial growth rate relative to the effluents (Fig 8). These results clearly indicated that the toxicity of the influent was significantly decreased after treatment in the packed-bed reactor at all HRTs tested. Additionally, it should be noted from Fig 8 that the gram-positive bacteria *E*. *faecalis* was more sensitive to MTBE and/or its degradation metabolites as compared to the gram-negative bacteria tested (*p* < 0.05).

Additionally, we observed that the maximum biomass concentration was reached in the stationary growth phase of all bacterial strains tested, occurring at 18 h for *P*. *aeruginosa* and 48 h for *E*. *coli*, *E*. *faecalis* and *K*. *pneumonia* (Table 2). Statistical analysis of the toxicity data indicated that the EC_50_ value for MTBE was higher for *E*. *coli*, *P*. *aeruginosa*, and *K*. *pneumoniae* (165.97 ± 40.1 mg L^-1^) relative to that of *E*. *faecalis* (88.71 ± 3.02 mg L^-1^) (Table 2), confirming that *E*. *coli*, *P*. *aeruginosa* and *K*. *pneumoniae* were less sensitive than *E*. *faecalis* to MTBE and/or MTBE-degradation metabolites present in the effluents from the reactor.

**Table 2:** EC_50_ values obtained in the microbial growth inhibition tests.

**Test bacteria**	**Time required to reach stationary growth phase [h]**	**EC**_**50**_ **[mg L**^**-1**^**]**
*E*. *coli* ATCC 25992	48	165.97 ± 40.1
*P*. *aeruginosa* ATCC 27853	18	165.97 ± 40.1
*E*. *faecalis* ATCC 29212	48	88.71 ± 3.02
*K*. *pneumoniae* ATCC 700603	48	165.97 ± 40.1

Furthermore, phytotoxicity bioassays were also performed using *Lactuca sativa* seeds as biological test organisms. Toxicity tests using plant species allow the assessment of adverse effects on seed germination and seedling development during the first days of growth. This type of bioassay allows assessment of potential adverse effects on plants caused by the discharge of contaminated water over soil [39].

The results on relative germination percent (RG%), relative growth index (RGI) and germination index (GI) of *L*. *sativa* of influent and effluents samples from the packed-bed reactor are reported in Table 3. It is evident that the RG%, RGI and GI values for the influent were much lower than for the effluents obtained at the different hydraulic retention times tested, which indicates that influent samples were much more inhibitory for *L*. *sativa* than effluent samples.

**Table 3:** Relative germination percentage (RG%), relative growth index (RGI) and germination index (GI) averages and toxicity categories of influent and effluent samples.

Parameter	Influent	Control	Hydraulic retention time [h]						
			62.6	17.1	13.8	7.5	5.8	5.4	4.1
**RG%**	45.33± 4.6	100	98.55±2.51	98.55±2.51	97.10±5.02	94.20±2.51	94.20±2.51	94.20±2.34	84.06±2.51
**RGI (%)**	54.815±6.9 (I)	100 (NSE)	89.87±10.66 (NSE)	88.76±11.2 (NSE)	83.63±6.05 (NSE)	83.55±5.78 (NSE)	83.41±5.66 (NSE)	77.79±6.33 (I)	72.00±5.69 (I)
**GI (%)**	31.56±11.4 (HPP)	100 (NP)	88.73±12.47 (NP)	88.33±13.1 (NP)	82.58±11.2 (NP)	80.00±8.73 (NP)	80.00±8.73 (NP)	75.18±10.2 (MPP)	63.86±9.81 (MPP)

Toxicity categories: I, inhibition of root elongation; NSE, no significant effects on root elongation; HPP, high presence of phytotoxic compounds; NP, no or low presence of phytotoxic compounds; MPP, moderate presence of phytotoxic compounds.

No significant differences (*p* < 0.05) were observed between the RG% values of the effluents samples obtained at HRTs ranging from 5.4 to 62.6 h and that of the control. In contrast, the RGI and GI values decreased as the HRT decreased, suggesting that higher MTBE and COD concentrations in the effluents from the bioreactor resulted in lower RGI and GI values. However, the results of the toxicity tests with *L*. *sativa* were acceptable according to the criteria established by Young et al. [39] and Zucconi et al. [40] because the RGI (≥ 80%) and GI (≥ 80%) values of the effluents obtained at HRTs ranging from 5.8 to 62.6 h indicated that most of the toxic components initially present in the medium supplied to the packed-bed reactor were eliminated or transformed into compounds not detrimental to *L*. *sativa* (i.e., the effluents did not induce significant adverse effects on *L*. *sativa*). However, the effluents obtained at HRTs of 4.1 and 5.4 h exhibited inhibitory effects on *L*. *sativa*, and the RGI and GI values of influent samples were 54.8% and 31.56%, indicating that they were highly toxic to *L*. *sativa*.

The above results indicated significantly reduced toxicity in the effluents from the packed-bed bioreactor following treatment and demonstrated that the microorganisms were more sensitive to MTBE and MTBE-degradation metabolites compared to *L*. *sativa*.

These results suggested that the lower HRT (5.8 h) necessary for the effective and efficient operation of the packed-bed reactor resulted in high MTBE and COD removal efficiencies and rates, as well as less toxic effluents obtained relative to the influent.

From the above mentioned, it is evident that the packed-bed biofilm reactor system proposed in our work exhibited superior characteristics to those in existence, for example very long operation periods, very high MTBE removal rates and efficiencies at very high MTBE loading rates, use of a novel, cheap and readily available support material, a small total reactor volume; all of which would reduce the capital and operation costs of the treatment process. In addition, the packed-bed reactor’s effluents are significantly less toxic, when compared to the influent.

## Conclusions

The lab-scale, packed-bed biofilm reactor used in this study efficiently degraded high MTBE concentrations (750 mg L^-1^) at HRTs from 5.8 to 62.6 h. The toxicity tests using organisms from two different trophic levels indicated that the effluent toxicity from the packed-bed reactor was significantly reduced following treatment. Additionally, our results indicated that the packed-bed reactor maintained its functional and operational stability over a wide range of HRTs and therefore may be a promising alternative to existing technologies for the biological removal of MTBE from high-strength MTBE-polluted water.
